# Sex-specific prognostic value of automated epicardial adipose tissue quantification on serial lung cancer screening chest computed tomography

**DOI:** 10.1093/ehjci/jeaf257

**Published:** 2025-08-29

**Authors:** Jan M Brendel, Thomas Mayrhofer, Ibrahim Hadzic, Emilia Norton, Isabel L Langenbach, Marcel C Langenbach, Matthias Jung, Vineet K Raghu, Konstantin Nikolaou, Pamela S Douglas, Michael T Lu, Hugo J W L Aerts, Borek Foldyna

**Affiliations:** Cardiovascular Imaging Research Center (CIRC), Department of Radiology, Massachusetts General Hospital, Harvard Medical School, 165 Cambridge Street Suite 400, Boston, MA 02114, USA; Department of Radiology, University of Tuebingen, Tuebingen, Germany; Center for Preventive Medicine and Digital Health, University of Heidelberg, Mannheim, Germany; Cardiovascular Imaging Research Center (CIRC), Department of Radiology, Massachusetts General Hospital, Harvard Medical School, 165 Cambridge Street Suite 400, Boston, MA 02114, USA; Radiology and Nuclear Medicine, CARIM & GROW, Maastricht University, Maastricht, The Netherlands; Cardiovascular Imaging Research Center (CIRC), Department of Radiology, Massachusetts General Hospital, Harvard Medical School, 165 Cambridge Street Suite 400, Boston, MA 02114, USA; Cardiovascular Imaging Research Center (CIRC), Department of Radiology, Massachusetts General Hospital, Harvard Medical School, 165 Cambridge Street Suite 400, Boston, MA 02114, USA; Department of Radiology, University of Cologne, Cologne, Germany; Cardiovascular Imaging Research Center (CIRC), Department of Radiology, Massachusetts General Hospital, Harvard Medical School, 165 Cambridge Street Suite 400, Boston, MA 02114, USA; Department of Radiology, University of Augsburg, Augsburg, Germany; Cardiovascular Imaging Research Center (CIRC), Department of Radiology, Massachusetts General Hospital, Harvard Medical School, 165 Cambridge Street Suite 400, Boston, MA 02114, USA; Cardiovascular Imaging Research Center (CIRC), Department of Radiology, Massachusetts General Hospital, Harvard Medical School, 165 Cambridge Street Suite 400, Boston, MA 02114, USA; Artificial Intelligence in Medicine (AIM) Program, Mass General Brigham, Harvard Medical School, 77 Avenue Louis Pasteur, Boston, MA 02115, USA; Department of Radiology, University of Tuebingen, Tuebingen, Germany; Duke Clinical Research Institute, Duke University School of Medicine, Durham, NC, USA; Cardiovascular Imaging Research Center (CIRC), Department of Radiology, Massachusetts General Hospital, Harvard Medical School, 165 Cambridge Street Suite 400, Boston, MA 02114, USA; Artificial Intelligence in Medicine (AIM) Program, Mass General Brigham, Harvard Medical School, 77 Avenue Louis Pasteur, Boston, MA 02115, USA; Radiology and Nuclear Medicine, CARIM & GROW, Maastricht University, Maastricht, The Netherlands; Artificial Intelligence in Medicine (AIM) Program, Mass General Brigham, Harvard Medical School, 77 Avenue Louis Pasteur, Boston, MA 02115, USA; Department of Radiation Oncology, Brigham and Women’s Hospital, Dana-Farber Cancer Institute, Harvard Medical School, Boston, MA, USA; Cardiovascular Imaging Research Center (CIRC), Department of Radiology, Massachusetts General Hospital, Harvard Medical School, 165 Cambridge Street Suite 400, Boston, MA 02114, USA; Artificial Intelligence in Medicine (AIM) Program, Mass General Brigham, Harvard Medical School, 77 Avenue Louis Pasteur, Boston, MA 02115, USA

**Keywords:** epicardial fat, inflammation, sex difference, women, low-dose, mortality risk

## Abstract

**Aims:**

Epicardial adipose tissue (EAT) is a metabolically active fat depot associated with coronary atherosclerosis and cardiovascular (CV) risk. While EAT is a known prognostic marker in lung cancer screening, its sex-specific prognostic value remains unclear. This study investigated sex differences in the prognostic utility of serial EAT measurements on low-dose chest computed tomography (CT).

**Methods and results:**

We analysed baseline and 2-year changes in EAT volume and density using a validated automated deep-learning algorithm in 24 008 heavy-smoking participants from the National Lung Screening Trial (NLST). Sex-stratified multivariable Cox models, adjusted for CV risk factors, body mass index (BMI), and coronary artery calcium (CAC), assessed associations between EAT and all-cause and CV mortality [median follow-up 12.3 years (IQR: 11.9–12.8), 4668 (19.4%) all-cause deaths, 1083 (4.5%) CV deaths]. Women (*n* = 9841; 41%) were younger, with fewer CV risk factors, lower BMI, fewer pack-years, and lower CAC than men (all *P* < 0.001). Baseline EAT was associated with similar all-cause and CV mortality risk in both sexes [max. aHR women: 1.70; 95% confidence interval (CI): 1.13–2.55; men: 1.83; 95% CI: 1.40–2.40, *P*-interaction = 0.986]. However, 2-year EAT changes predicted CV death only in women (aHR: 1.82; 95% CI: 1.37–2.49; *P* < 0.001), and showed a stronger association with all-cause mortality in women (aHR: 1.52; 95% CI: 1.31–1.77) than in men (aHR: 1.26; 95% CI: 1.13–1.40; *P*-interaction = 0.041).

**Conclusion:**

In this large lung cancer screening cohort, serial EAT changes independently predicted CV mortality in women and were more strongly associated with all-cause mortality in women than in men. These findings support routine EAT quantification on chest CT for improved sex-specific cardiovascular risk stratification.

## Graphical Abstract

**Graphical Abstract:**
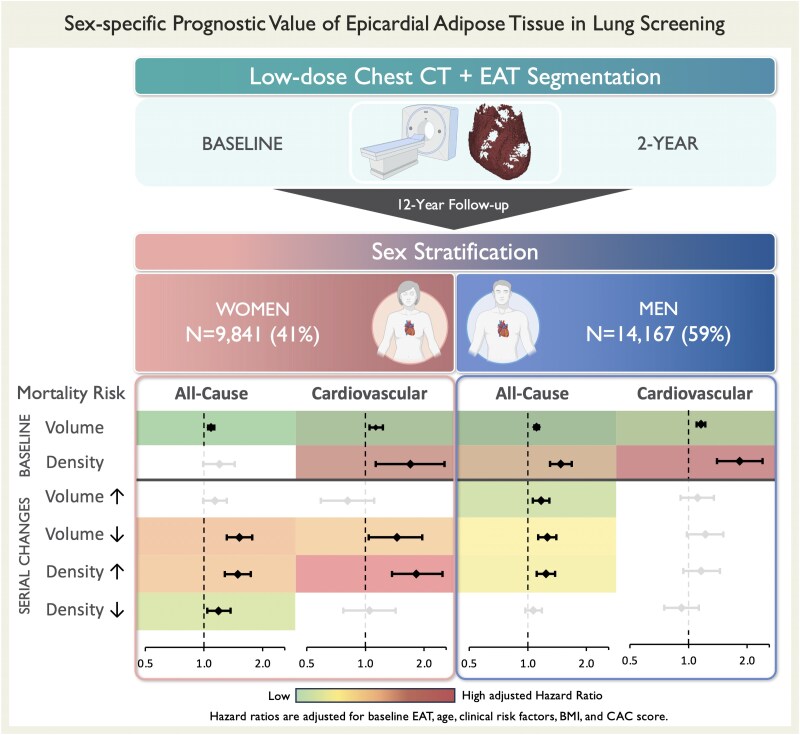
Epicardial adipose tissue (EAT) and its longitudinal change predict mortality in women and men undergoing lung cancer screening. EAT changes predicted cardiovascular mortality only in women and showed stronger associations with all-cause mortality in women than in men. Automated EAT quantification may enhance cardiovascular risk stratification, particularly for women. Created in BioRender.^1^

**See the editorial comment for this article 'Advanced cardiac imaging in primary prevention: the great challenge we have to achieve', by E. Conte**  ***et al*****., https://doi.org/10.1093/ehjci/jeaf268.**

## Introduction

Lung cancer remains the leading cause of cancer-related death worldwide in both women and men.^2^ Its incidence is rising in women and approaches or even exceeds that of men in several countries, suggesting an increasing future burden among women.^3,4^ Low-dose computed tomography (LDCT) screening reduces mortality by at least 20% in high-risk individuals, with evidence of greater benefit in women.^5–7^ Several countries, including the USA, Canada, Australia, China, and South Korea, have implemented LDCT lung cancer screening, with growing momentum across Europe.^8^

While smoking is the primary risk factor for lung cancer, it also substantially increases cardiovascular disease (CVD) risk, with smokers more likely to die from CVD than from lung cancer.^5,7,9^ Importantly, LDCT scans cover the heart and thereby provide a unique opportunity to evaluate both pulmonary and cardiovascular (CV) health. In this context, LDCT scans allow for opportunistic quantification of epicardial adipose tissue (EAT), a metabolically active fat depot surrounding the heart that promotes atherosclerosis and heart failure and has been associated with both all-cause and CV mortality.^10–16^

However, it remains unclear whether the prognostic value of EAT differs by sex. We hypothesized that serial EAT measurements on LDCT scans would be differently associated with all-cause and CV mortality in women and men. Given the underestimation of CV risk in women,^17^ understanding sex-related differences in EAT may improve personalized management in this high-risk population. Thus, we aimed to evaluate the sex-specific prognostic value of EAT in a large primary prevention cohort undergoing lung cancer screening.

## Methods

### Study setting and participants

We included current and former smokers aged 55–74 years from the National Lung Screening Trial (NLST), a community-based, randomized controlled trial that enrolled individuals between 2002 and 2004 at 33 sites across the USA (ClinicalTrials.gov identifier: NCT00047385).^5,18^ Eligible participants were current or former smokers with a minimum smoking history of 30 pack-years and no prior lung cancer diagnosis, signs, or symptoms. LDCT arm participants were invited to annual screening sessions, and we included all participants with evaluable baseline scans for baseline EAT analysis. To assess longitudinal EAT changes, we included those with additional 2-year follow-up LDCT scans. Participants with missing or corrupted clinical or imaging data were excluded (see Supplementary data online, *Figure S1*).

All subjects were followed prospectively for a median of 12.3 years (IQR: 11.9–12.8) for adverse events, including all-cause mortality and CV mortality, defined as fatal myocardial infarction or stroke; mortality endpoints were centrally adjudicated through the NLST Endpoint Verification Process, which systematically reviewed and validated causes of death.^5^ The NLST data have been approved for secondary use by the National Cancer Institute and the local Institutional Review Board. Written consent was obtained for the parent trial, and a waiver of consent was granted for this retrospective study.

### LDCT image acquisition and EAT measurements

**Figure 1:**
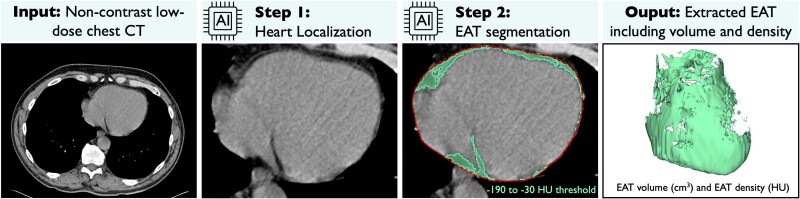
Overview of the deep-learning framework for EAT quantification. Deep-learning–based EAT segmentation on low-dose lung cancer screening CT. EAT was segmented from non-contrast, low-dose chest CT scans using a fully automated deep learning framework. The process involved heart localization, pericardial sac segmentation (red line), and extraction of epicardial fat (green) based on predefined attenuation thresholds (−190 to −30 HU). HU, Hounsfield units; EAT, epicardial adipose tissue; CT, computed tomography.

Chest LDCT scans were acquired using multi-detector CT scanners with non-electrocardiogram (ECG)-synchronized, non-contrast protocols.^5,18^ EAT volume was quantified using a validated, fully automated, open-source deep-learning algorithm, which performs three sequential steps: heart localization, pericardial sac segmentation using 3D U-Net architecture, and EAT rendering based on voxels within the pericardium with attenuation between −190 and −30 Hounsfield units (HU) (*Figure 1*).^13,15,19^ The primary variables of interest were EAT volume (cm^3^) and density (HU). To account for individual differences in body habitus, absolute EAT volumes were indexed to body surface area (BSA; m^2^). EAT density was determined as the mean attenuation of all segmented EAT voxels for each individual. Changes in EAT volume and density were calculated by subtracting baseline measurements from follow-up values.

### Statistical analysis

Continuous data are presented as means ± standard deviations or medians with interquartile ranges (Q1–Q3), while categorical data are shown as absolute and relative frequencies. Baseline characteristics were stratified by sex and compared using independent two-sample *t*-tests, Wilcoxon rank-sum tests or Fisher’s exact tests.

We identified sex-specific empirical cut-off values for typical and atypical changes in EAT (i.e. increases and decreases), using a data-driven receiver operating characteristic (ROC) approach to predict all-cause and CV mortality. Women and men were then divided into groups based on relative EAT changes (% = [(EAT₂ − EAT₁)/EAT₁] × 100), with empirical cut-offs calculated by minimizing the Euclidean distance between the ROC curve and the point of perfect sensitivity and specificity. These cut-offs delineated the typical ranges of EAT changes in each sex.

Crude mortality rates of women and men were calculated based on baseline EAT values (above or below the sex-specific median) and EAT changes (stable, increase, and decrease). Cox proportional hazards regression models were used to calculate hazard ratios (HRs) and 95% confidence intervals (CIs) for associations between EAT and mortality. Multivariable models were adjusted for EAT volume and density and CV risk factors [age, sex, race, ethnicity, smoking status (former vs. current), pack-years, history of heart disease, myocardial infarction [MI], or stroke, diabetes, hypertension, education status, body mass index (BMI), and coronary artery calcium (CAC) score]. A sensitivity analysis was done to investigate the effect of technical imaging parameters [slice thickness, tube voltage (kVp), tube current–exposure time product (mAs), and signal-to-noise ratio] on EAT’s prognostic value in women and men. Variance inflation factor calculation through linear regression models showed no evidence of multicollinearity for EAT volume and density.

Kaplan–Meier curves and log-rank tests compared cumulative event rates across baseline EAT values and EAT changes. Cox-restricted cubic spline regression analyses were performed to depict non-linear associations between absolute changes in EAT volume or density and mortality. Given the study’s exploratory nature, inferences were guided by a two-sided 5% false-positive error rate without adjustment for multiple comparisons. All statistical analyses were performed using Stata 17.0 (StataCorp LP, College Station, Texas).

## Results

### Study population

The study included 24 008 NLST participants who underwent LDCT at baseline. Of these, 86% received a 2-year follow-up LDCT and were eligible for assessment of EAT changes.

At baseline, 9841 participants (41%) were women with a mean age of 61 ± 5 years, while 14 167 participants (59%) were men with a mean age of 62 ± 5 years. Despite a higher proportion of current smokers (women vs. men; 50% vs. 46%), women had a lower median pack-year history [44 (38–60) vs. 52 (41–72)]. They also had fewer traditional CV risk factors such as diabetes (7% vs. 11%), hypertension (34% vs. 36%), and half the history of heart disease or MI (8% vs. 17%). Women also had lower CAC scores [median 13 (IQR: 0–147) vs. median 140 (IQR: 10–585)] and a slightly lower BMI (27.4 ± 5.6 kg/m^2^ vs. 28.2 ± 4.6 kg/m^2^; *P* < 0.001 for all). Detailed baseline characteristics are provided in *Table 1* and Supplementary data online, *Table S1*.

**Table 1:** Baseline characteristics stratified by sex

	All (*N* = 24 008)	Women (*n* = 9841)	Men (*n* = 14 167)	*P*
Age, years	61.4 ± 5.0	61.2 ± 4.9	61.6 ± 5.1	**<0**.**001**
Race				**<0**.**001**
Caucasian	21 888 (91.2)	9031 (91.8)	12 857 (90.8)	
African American	1042 (4.3)	470 (4.8)	572 (4.0)	
Asian	521 (2.2)	124 (1.3)	397 (2.8)	
Other/unknown	557 (2.3)	216 (2.2)	341 (2.4)	
Ethnicity				0.048
Hispanic/Latinx	428 (1.8)	151 (1.5)	277 (2.0)	
Non-Hispanic/Latinx	23 486 (97.8)	9653 (98.1)	13 833 (97.6)	
Other/unknown	94 (0.4)	37 (0.4)	57 (0.4)	
Smoking				**<0**.**001**
Former	12 482 (52.0)	4893 (49.7)	7589 (53.6)	
Current	11 526 (48.0)	4948 (50.3)	6578 (46.4)	
Pack-years	48 (39–66)	44 (38–60)	52 (41–72)	**<0**.**001**
History of heart disease or MI	3086 (12.9)	747 (7.6)	2339 (16.5)	**<0**.**001**
History of stroke	656 (2.7)	265 (2.7)	391 (2.8)	0.778
Diabetes mellitus	2317 (9.7)	709 (7.2)	1608 (11.4)	**<0**.**001**
Hypertension	8438 (35.2)	3322 (33.8)	5116 (36.1)	**<0**.**001**
Level of education				**<0**.**001**
High school graduate or below	6999 (29.2)	3247 (33.0)	3752 (26.5)	
Post-high school (excluding college)	3373 (14.1)	1427 (14.5)	1946 (13.7)	
Some college or bachelor’s degree	9691 (40.4)	3761 (38.2)	5930 (41.9)	
Graduate school	3475 (14.5)	1174 (11.9)	2301 (16.2)	
Other/unknown	470 (2.0)	232 (2.4)	238 (1.7)	
BMI, kg/m²	27.9 ± 5.0	27.4 ± 5.6	28.2 ± 4.6	**<0**.**001**
BSA, m²	1.99 ± 0.25	1.82 ± 0.21	2.10 ± 0.21	**<0**.**001**
LDCT measures				
EAT volume (BSA-indexed), cm³/m²	70.3 ± 24.6	65.7 ± 23.2	73.5 ± 25.0	**<0**.**001**
	67.4 [52.8–84.8]	62.6 [49.1–79.2]	70.8 [55.9–88.0]	
EAT density, HU	−77.7 ± 5.2	−77.2 ± 5.1	−78.0 ± 5.2	**<0**.**001**
	−77.6 [−81.2– −74.0]	−77.0 [−80.7– −73.6]	−78.0 [−81.5– −74.4]	
CAC score^a^, Agatston units	61.7 (1.25–376)	13.0 (0.00–147)	140 (9.70–585)	**<0**.**001**
All-cause mortality	4668 (19.4)	1532 (15.6)	3136 (22.1)	**<0**.**001**
CV mortality	1083 (4.5)	323 (3.3)	760 (5.4)	**<0**.**001**

Values are given as mean ± standard deviation, median (Q1–Q3), or raw number (percentage). *P*-values in bold indicate statistical significance (*P* < 0.05). BMI, body mass index; BSA, body surface area; CAC, coronary artery calcium; CV, cardiovascular; EAT, epicardial adipose tissue; HU, Hounsfield units, MI, myocardial infarction.

^a^CAC score was available in 5721 women and 8193 men.

All-cause mortality rates were 16% (1532/9841) in women and 22% (3136/14 167) in men (see Supplementary data online, *Table S2*). CV mortality rates were 3% (323/9841) in women and 5% (760/14 167) in men.

### Sex differences in baseline EAT

At baseline, women had lower mean EAT volumes than men (65.7 ± 23.2 cm³/m² vs. 73.5 ± 25.0 cm³/m²; *P* < 0.001) (*Table 1*). Baseline EAT density was slightly higher (less negative) in women than men (−77.2 ± 5.1 HU vs. −78.0 ± 5.2 HU; *P* < 0.001). EAT density was inversely related to EAT volume in women and men (both *r* = −0.75; *P* < 0.001).

**Figure 2:**
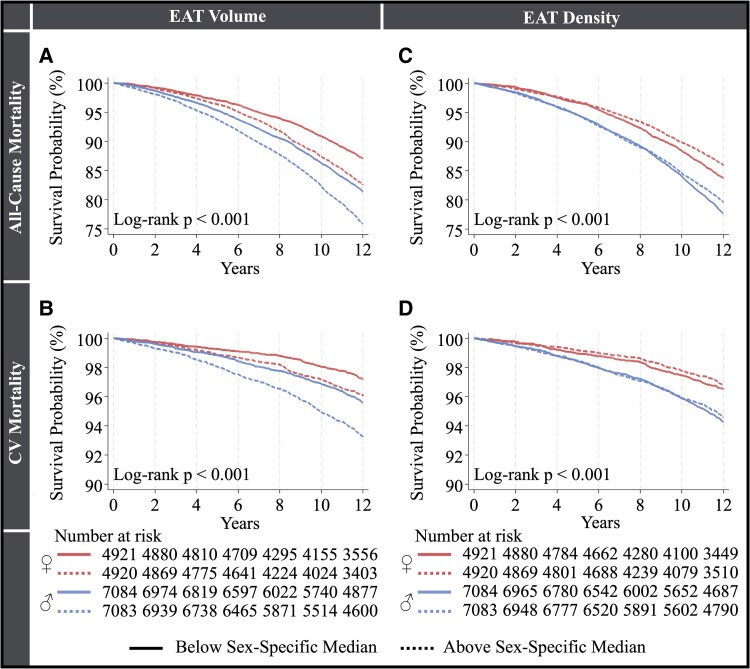
Baseline EAT vs. mortality in women and men. Kaplan–Meier survival curves show sex-specific associations of baseline EAT volume (*A* and *B*) and density (*C* and *D*) with all-cause and cardiovascular mortality. CV, cardiovascular; EAT, epicardial adipose tissue.

In univariable analysis, EAT volume was associated with a 9–13% higher mortality risk per 10 cm³/m² in both sexes (*P*-interaction = 0.125 for all-cause mortality and *P*-interaction = 0.707 for CV mortality) (*Table 2*). The corresponding Kaplan–Meier survival curves are displayed in *Figure 2*.

**Table 2:** Association of baseline EAT with mortality in women and men

	EAT volume							EAT density						
	Women			Men			Interaction	Women			Men			Interaction
	HR	95% Cl	*P*	HR	95% Cl	*P*	P*	HR	95% Cl	*P*	HR	95% Cl	*P*	P*
All-cause mortality														
Univariable	1.11	1.09–1.13	**<0**.**001**	1.09	1.07–1.10	**<0**.**001**	0.125	0.82	0.75–0.91	**<0**.**001**	0.93	0.87–1.00	0.051	**0**.**038**
Model 1	1.17	1.14–1.21	**<0**.**001**	1.18	1.16–1.20	**<0**.**001**	0.102	1.45	1.25–1.67	**<0**.**001**	1.73	1.56–1.91	**<0**.**001**	**0**.**010**
Model 2	1.10	1.07–1.13	**<0**.**001**	1.11	1.08–1.13	**<0**.**001**	0.524	1.28	1.10–1.47	**0**.**001**	1.41	1.28–1.56	**<0**.**001**	0.260
Model 3	1.09	1.05–1.13	**<0**.**001**	1.11	1.08–1.14	**<0**.**001**	0.832	1.20	0.99–1.44	0.063	1.48	1.30–1.68	**<0**.**001**	0.209
Cardiovascular mortality														
Univariable	1.13	1.08–1.18	**<0**.**001**	1.12	1.09–1.15	**<0**.**001**	0.707	0.89	0.72–1.10	0.294	0.93	0.81–1.06	0.277	0.764
Model 1	1.25	1.18–1.32	**<0**.**001**	1.25	1.20–1.30	**<0**.**001**	0.690	1.97	1.45–2.67	**<0**.**001**	2.16	1.76–2.64	**<0**.**001**	0.530
Model 2	1.13	1.06–1.20	**<0**.**001**	1.16	1.11–1.21	**<0**.**001**	0.805	1.76	1.29–2.41	**<0**.**001**	1.78	1.45–2.19	**<0**.**001**	0.876
Model 3	1.13	1.05–1.23	**0**.**002**	1.16	1.10–1.22	**<0**.**001**	0.798	1.70	1.13–2.55	**0**.**010**	1.83	1.40–2.40	**<0**.**001**	0.986

Model 1, EAT volume and density; Model 2, Model 1 + age, race, ethnicity, smoking (current vs. former), pack-years, history of heart disease or MI, history of stroke, diabetes mellitus, hypertension, education status, and BMI; and Model 3, Model 2 + CAC score.

Hazard ratios (HRs and 95% CIs are per 10 cm^3^/m^2^ (EAT volume) or 10 HU (EAT density).

*P*-values in bold indicate statistical significance (*P* < 0.05). P* = *P*-value for sex as interaction term.

BMI, body mass index; CAC, coronary artery calcium; EAT, epicardial adipose tissue; HU, Hounsfield units.

Associations remained significant after multivariable adjustment for both all-cause mortality (aHR: 1.09; 95% CI: 1.05–1.13; *P* < 0.001 for women and aHR: 1.11; 95% CI: 1.08–1.14, *P* < 0.001 for men; *P*-interaction = 0.832) and CV mortality (aHR: 1.13; 95% CI: 1.05–1.23, *P* = 0.002 for women vs. aHR: 1.16; 95% CI: 1.10–1.22 for men; *P* < 0.001; *P*-interaction = 0.789) (*Table 2*). In both sexes, baseline EAT density was associated with increased CV mortality (aHR: 1.70, 95% CI: 1.13–2.55 per 10 HU for women vs. aHR: 1.83, 95% CI: 1.40–2.40 for men; *P*-interaction = 0.986). Technical parameters did not affect these associations as detailed in Supplementary data online, *Table S3*.

### Sex differences in EAT changes

**Figure 3:**
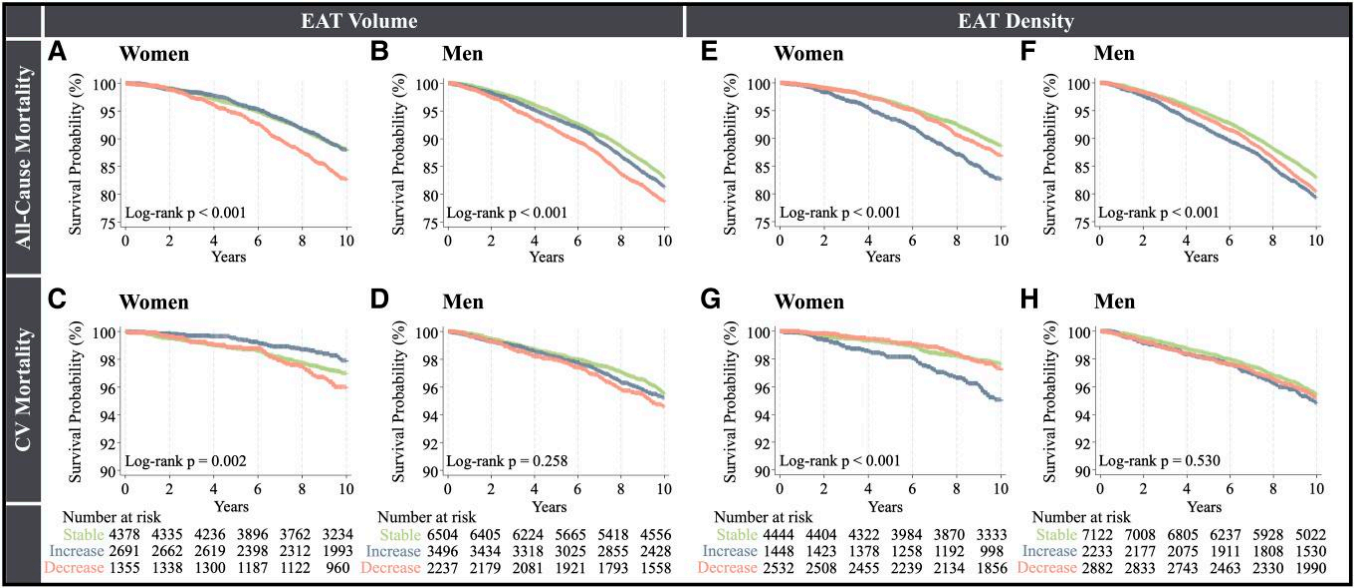
Eat changes vs. mortality in women and men. Kaplan–Meier survival curves illustrate sex-specific associations of 2-year increases or decreases in EAT volume (*A–D*) and density (*E–H*) with all-cause and cardiovascular mortality in women and men. EAT changes are categorized as stable (green), increased (blue), and decreased (orange). Stable EAT volume ranged from −7.5% to +10.4% in women and from −7.6% to +11.1% in men. Stable EAT density ranged from −2.5% to +2.3% in women and from −3.2% to +2.5% in men. CV, cardiovascular; EAT, epicardial adipose tissue.

Over 2 years, the mean EAT volume increased with similar magnitude in both sexes (+2.5 ± 10.2 cm³/m² in women vs. + 2.5 ± 11.6 cm³/m² in men; *P* = 0.850) (see Supplementary data online, *Table S4*). Meanwhile, EAT density decreased in both sexes, with a slightly greater decrease in women than in men (−0.6 ± 2.8 HU vs. −0.5 ± 3.1 HU; *P* = 0.005). Stable EAT volume (stable category ranges: −7.5% to +10.4% for women, −7.6% to +11.1% for men) was seen in a similar proportion of women and men (52% vs. 53%; *P* = 0.095). Stable EAT density (stable category ranges: −2.5% to +2.3% for women, −3.2% to +2.5% for men) was less frequent in women than in men (53% vs. 58%; *P* < 0.001). Women were also more likely than men to experience a decrease in EAT density (30% vs. 24%; *P* < 0.001). *Figure 3* displays Kaplan–Meier survival curves for women and men, categorized by stable, increased, or decreased EAT.

A decrease in EAT volume was associated with all-cause mortality more strongly in women than in men (aHR: 1.52; 95% CI: 1.31–1.77 vs. aHR: 1.26; 95% CI: 1.13–1.40; *P*-interaction = 0.041) and was associated with CV mortality exclusively in women (aHR: 1.45; 95% CI: 1.95–1.99; *P* = 0.022) (*Table 3*). EAT volume increase was linked to all-cause mortality only in men (aHR: 1.17; 95% CI: 1.06–1.29; *P* = 0.001).

**Table 3:** Association of EAT change with mortality in women and men

		EAT volume							EAT density						
		Women			Men			Interaction	Women			Men			Interaction
		HR	95% Cl	*P*	HR	95% Cl	*P*	P*	HR	95% Cl	*P*	HR	95% Cl	*P*	P*
**All-cause mortality**															
** Univariable**	Stable	Ref			Ref				Ref			Ref			
	Increase	1.02	0.89–1.16	0.802	1.13	1.02–1.24	**0**.**014**	0.231	1.59	1.37–1.84	**<0**.**001**	1.23	1.11–1.37	**<0**.**001**	**0**.**006**
	Decrease	1.49	1.29–1.74	**<0**.**001**	1.28	1.15–1.42	**<0**.**001**	0.096	1.21	1.06–1.39	**0**.**005**	1.19	1.08–1.31	**<0**.**001**	0.836
** Model 1**	Stable	Ref			Ref				Ref			Ref			
	Increase	1.11	0.97–1.28	0.129	1.22	1.11–1.34	**<0**.**001**	0.235	1.58	1.36–1.84	**<0**.**001**	1.27	1.14–1.41	**<0**.**001**	**0**.**006**
	Decrease	1.44	1.24–1.68	**<0**.**001**	1.25	1.13–1.39	**<0**.**001**	0.124	1.19	1.04–1.37	**0**.**013**	1.10	1.00–1.22	0.052	0.706
** Model 2**	Stable	Ref			Ref				Ref			Ref			
	Increase	1.14	0.99–1.31	0.068	1.17	1.06–1.29	**0**.**001**	0.779	1.50	1.28–1.74	**<0**.**001**	1.23	1.10–1.36	**<0**.**001**	**0**.**019**
	Decrease	1.51	1.30–1.76	**<0**.**001**	1.24	1.11–1.37	**<0**.**001**	**0**.**027**	1.19	1.04–1.37	**0**.**014**	1.07	0.97–1.19	0.162	0.354
** Model 3**	Stable	Ref			Ref				Ref			Ref			
	Increase	1.14	0.99–1.31	0.064	1.17	1.06–1.29	**0**.**001**	0.820	1.49	1.28–1.74	**<0**.**001**	1.24	1.11–1.38	**<0**.**001**	**0**.**025**
	Decrease	1.52	1.31–1.77	**<0**.**001**	1.26	1.13–1.40	**<0**.**001**	**0**.**041**	1.19	1.04–1.37	**0**.**014**	1.07	0.97–1.18	0.193	0.321
**Cardiovascular mortality**															
** Univariable**	Stable	Ref			Ref				Ref			Ref			
	Increase	0.71	0.52–0.97	**0**.**032**	1.05	0.86–1.27	0.637	**0**.**038**	1.95	1.44–2.65	**<0**.**001**	1.11	0.89–1.38	0.353	**0**.**003**
	Decrease	1.37	1.00–1.88	**0**.**049**	1.20	0.97–1.48	0.100	0.485	1.12	0.83–1.52	0.446	1.09	0.90–1.33	0.384	0.877
** Model 1**	Stable	Ref			Ref				Ref			Ref			
	Increase	0.79	0.57–1.07	0.130	1.16	0.96–1.41	0.130	**0**.**039**	1.99	1.46–2.70	**<0**.**001**	1.17	0.94–1.45	0.168	**0**.**003**
	Decrease	1.32	0.96–1.81	0.086	1.16	0.94–1.44	0.164	0.553	1.06	0.78–1.45	0.691	0.96	0.78–1.17	0.681	0.767
** Model 2**	Stable	Ref			Ref				Ref			Ref			
	Increase	0.81	0.59–1.11	0.184	1.10	0.90–1.33	0.361	0.112	1.81	1.33–2.47	**<0**.**001**	1.14	0.92–1.42	0.237	**0**.**016**
	Decrease	1.43	1.04–1.96	**0**.**028**	1.15	0.93–1.43	0.196	0.313	1.04	0.77–1.42	0.783	0.93	0.76–1.14	0.494	0.600
** Model 3**	Stable	Ref			Ref				Ref			Ref			
	Increase	0.81	0.59–1.11	0.198	1.11	0.91–1.35	0.311	0.129	1.82	1.37–2.49	**<0**.**001**	1.16	0.94–1.45	0.172	**0**.**018**
	Decrease	1.45	1.05–1.99	**0**.**022**	1.22	0.98–1.51	0.071	0.398	1.05	0.77–1.43	0.769	0.92	0.75–1.13	0.447	0.545

Model 1, adjusted for baseline EAT values; Model 2, Model 1 + age, race, ethnicity, smoking (current vs. former), pack-years, history of heart disease or MI, history of stroke, diabetes mellitus, hypertension, education status, and BMI; and Model 3, Model 2 + CAC score.

Patients with stable EAT functioned as reference groups. Stable EAT volume ranged from −7.5 to +10.4% in women and from −7.6 to +11.1% in men. Stable EAT density ranged from −2.5 to +2.3% and from −3.2 to +2.5% in men. EAT changes falling outside these sex-specific ranges were classified as atypical increases or decreases.

*P*-values in bold indicate statistical significance (*P* < 0.05). P* = *P*-value for sex as interaction term.

BMI, body mass index; CAC, coronary artery calcium; EAT, epicardial adipose tissue; HU, Hounsfield units.

**Figure 4:**
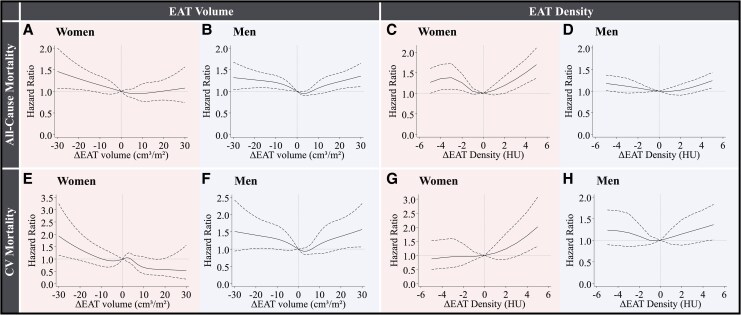
Sex-specific non-linear associations between EAT changes and mortality risk. Cox-restricted cubic spline curves show associations between changes in EAT over time with all-cause (*A–D*) and cardiovascular mortality (*E–H*). EAT volume increase was linked to a higher risk of all-cause mortality in men (*B*) but not in women (*A*). EAT density increase led to a steeper rise in all-cause mortality risk in women (*C*) than in men (*D*) and substantially elevated cardiovascular mortality risk in women (*G*). All hazard ratios are adjusted for baseline EAT, age, clinical risk factors, BMI, and CAC score. CV, cardiovascular; EAT, epicardial adipose tissue; HU, Hounsfield units.

An increase in EAT density was associated with all-cause mortality more strongly in women than in men (aHR: 1.49; 95% CI: 1.28–1.74 vs. aHR: 1.24; 95% CI: 1.11–1.38; *P*-interaction = 0.025) and was associated with CV mortality exclusively in women (aHR: 1.82; 95% CI: 1.37–2.49; *P* < 0.001), as shown in *Table 3*. EAT density decrease was linked to all-cause mortality only in women (aHR: 1.19; 95% CI: 1.04–1.37; *P* = 0.014). Associations between changes in EAT over time and mortality risk are illustrated using cubic spline curves in *Figure 4*; crude mortality rates are given in Supplementary data online, *Table S5*.

## Discussion

This study evaluated the sex-specific prognostic values of EAT in a large prospective cohort of asymptomatic individuals undergoing LDCT for lung cancer screening. We observed that baseline EAT was associated with a similar increase in the risk of all-cause and CV mortality among both women and men. In contrast, 2-year changes in EAT predicted CV death only in women and were twice as strongly associated with all-cause mortality in women as in men.

The NLST provided a robust dataset with serial LDCT scans, enabling comprehensive cross-sectional and longitudinal EAT analysis. Notably, the trial included almost 10 000 women and had a relatively balanced sex distribution (41% women), compared with other lung screening trials. Most other trials underrepresented women, with female participants constituting only 17–36% (NELSON, MILD, ITALUNG, DEPISCAN, and UKLS), while DANTE excluded women entirely. Only LUSI (50% women) and DLCST (44% women) had comparable sex distributions, though their smaller sample sizes may limit their generalizability.^20–26^

At baseline, women had a lower EAT volume than men (66 cm³/m² vs. 74 cm³/m²), reflecting known sex differences in fat distribution, composition, and cardiometabolic risk^27,28^ and underscoring the importance of accounting for sex when assessing EAT. A 10 cm^2^/m^2^ higher EAT volume at baseline was associated with an increased hazard of all-cause mortality (9% and 11%) and CV mortality (13% and 16%) in women and men, corroborating prior evidence linking EAT volume to adverse events across different populations.^15,29,30^ Investigating the changes in EAT over time, a mild increase in EAT volume was observed in women and men (both +2.5 cm³/m²). This can be explained, at least in part, by a general body fat increase and redistribution (increase in visceral fat and decrease in subcutaneous fat) with ageing.^31^ A decrease in EAT volume was linked to an elevated risk of all-cause mortality in both women (+52%) and men (+26%). Given the vulnerable cohort and increased risk of cancer, this may reflect underlying conditions, such as cancer-related cachexia. In contrast, an interval increase in EAT volume was associated with higher all-cause mortality only in men, possibly due to their greater susceptibility to metabolic disturbances from increased visceral fat.^32^

EAT density and volume exhibited a strong inverse correlation (*r* = −0.75), consistent with prior studies,^14,15^ underlining the importance of adjusting for EAT volume when assessing EAT density. Increasing EAT density may indicate fibrosis and inflammation rather than simple volumetric changes. In heavy smokers, this could reflect irreversible adipose dysfunction akin to metabolic syndrome-associated ectopic fat accumulation. Higher EAT density has been linked to systemic inflammation and cachexia in chronic disease, suggesting it may serve as both a biomarker and mediator of CV risk.^11,33,34^ Notably, we observed an association between an increase in EAT density over time and all-cause mortality in both sexes, with twice the effect in women (aHR: 1.49) compared with men (aHR: 1.24; *P*-interaction = 0.025). Further, women with an increase in EAT density had an 82% greater risk of CV mortality, an association exclusive to women (*P* < 0.001). This may suggest sex-specific pathophysiology, possibly linked to inflammation, as inflamed EAT undergoes changes secondary to lipolysis, inhibited lipogenesis, and perivascular oedema, resulting in greater EAT density on CT.^35^ Supporting this hypothesis, other studies have linked greater EAT density to elevated systemic proinflammatory biomarkers such as IL-6, IL-1β, MCP-1, and TNFα, atherogenesis, and ultimately heightened mortality risk.^10,34^

Furthermore, sex-specific differences in EAT may stem from hormonal influences, particularly oestrogen and testosterone, which regulate fat distribution and inflammation. Oestrogen limits visceral fat and local inflammation in premenopausal women, while its decline after menopause may lead to increased visceral fat, inflammation, and cardiovascular remodeling.^27,32^ Therefore, hormonal shifts likely explain EAT’s stronger prognostic value in our cohort of largely postmenopausal women. In men, testosterone helps maintain lean muscle mass and prevent ectopic fat accumulation, but its age-related decline may contribute to EAT volume increase over time. The lack of sex-specific imaging biomarkers in risk models may contribute to the underestimation of cardiovascular risk in women. Future studies should evaluate whether EAT-based risk stratification could improve personalized cardiovascular prevention, especially in postmenopausal women, in whom metabolic shifts heighten CV vulnerability. Prospective studies are warranted to evaluate the clinical relevance of EAT changes and to explore potential interventions aimed at modifying them.

### Limitations

We acknowledge several limitations. First, while key predictors such as age, sex, and BMI were included, lipid profiles and history of dyslipidaemia were not recorded in the NLST and therefore could not be included in our analysis. Secondly, the NLST cohort consisted exclusively of heavy smokers, predominantly White, limiting generalizability to more diverse populations, including non-smokers and those with lower smoking exposure. Thirdly, the retrospective nature of this study introduces methodological limitations, including the absence of certain covariates, such as steroid or statin use, menopausal status, and site-specific LDCT acquisition parameters. Finally, only 86% of NLST participants underwent a 2-year follow-up LDCT, limiting longitudinal assessment to this subset; additionally, categorizing EAT changes over time may have introduced misclassification bias, potentially affecting risk estimates.

### Clinical implications

Our findings emphasize the prognostic value of routine EAT monitoring, particularly in women. Women with changes in EAT density demonstrated a unique vulnerability to adverse events, underscoring the need for sex-specific prognostic strategies in cardiovascular prevention. With the expected rise in serial LDCT screenings and the number of women screened in the coming decades,^36^ automated EAT quantification offers a practical measurement approach. Integrating opportunistic EAT assessment into lung cancer screening could enhance risk stratification with targeted interventions, such as anti-inflammatory therapies (e.g. colchicine and IL-6 inhibitors) and metabolic treatments (e.g. GLP-1 receptor agonists and SGLT-2 inhibitors),^37–39^ which may be of greater benefit to women than to men.

## Conclusion

Serial EAT changes predicted cardiovascular mortality in women and were more strongly associated with all-cause mortality in women than in men. In particular, women exhibited a strong relationship between EAT density and cardiovascular mortality, suggesting a pathophysiology driven by inflammation and hormonal changes. In contrast, men showed an association between EAT volume and all-cause mortality, likely reflecting a greater metabolic vulnerability from visceral fat expansion. These findings underscore the need for sex-specific cardiovascular risk assessment. Automated EAT measurement in LDCT lung cancer screening could enhance personalized and sex-specific risk stratification and guide targeted prevention strategies.

## Supplementary Material

jeaf257_Supplementary_Data

## Data Availability

The datasets generated during and/or analysed during the current study are available from the corresponding author on reasonable request.
