# Supplementary material for: Sex-specific prognostic value of automated epicardial adipose tissue quantification on serial lung cancer screening chest computed tomography
Source: Eur Heart J Cardiovasc Imaging. 2025 Aug 29;26(11):1782–92. doi: 10.1093/ehjci/jeaf257 (PMC12571501; doi:10.1093/ehjci/jeaf257)
Supplement: jeaf257_Supplementary_Data [file jeaf257_supplementary_data.docx]

**Sex-Specific Prognostic Value of Automated Epicardial Adipose Tissue Quantification on Serial Lung Cancer Screening Chest CT**

**SUPPLEMENTAL MATERIAL**

**FIGURE S1: CONSORT Diagram**


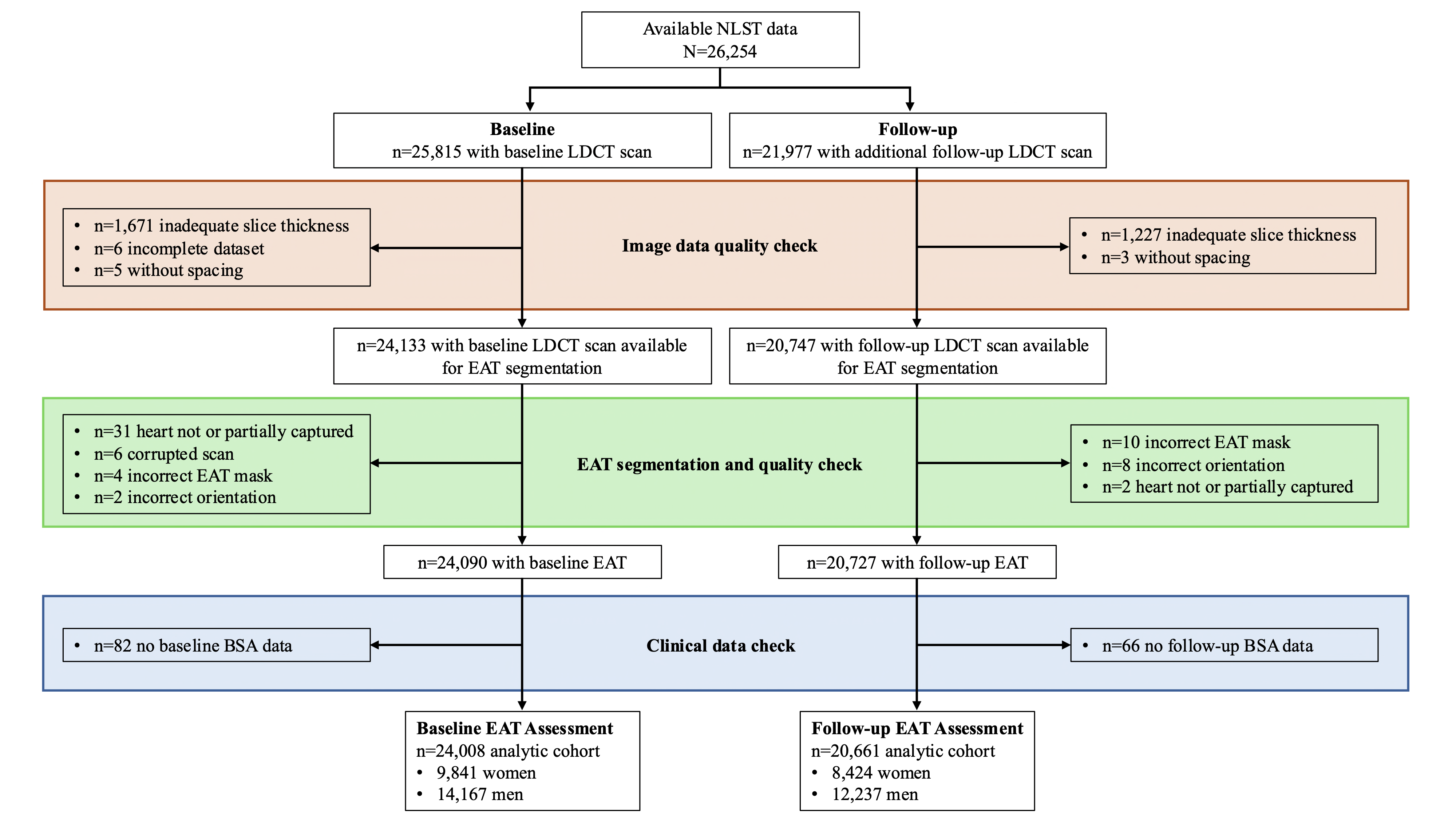


Flow-diagram displaying overview of the study cohort.

BSA = body surface area; EAT = epicardial adipose tissue; LDCT = low dose computed tomography; NLST = National Lung Screening Trial.

**TABLE S1. Additional Baseline Characteristics Stratified by Sex**

| Technical imaging parameter | All (N=24,008) | Women (n=9,841) | Men (n=14,167) |
| --- | --- | --- | --- |
| Slice thickness [mm] | 2.4 ± 0.7 | 2.4 ± 0.7 | 2.4 ± 0.7 |
| Tube voltage [kVp] | 120 [120–120] | 120 [120–120] | 120 [120–120] |
| Tube current – exposure time product [mAs] | 60.8 ± 26.4 | 58.9 ± 26.7 | 62.1 ± 26.0 |
| Signal-to-noise ratio | 2.4 ± 0.2 | 2.4 ± 0.2 | 2.4 ± 0.2 |

Values are given as mean ± standard deviation, or median (Q1–Q3).

**TABLE S2. Crude Mortality Rates Across Categories of Baseline EAT in Women and Men**

|  | Baseline EAT in Women | | |  | Baseline EAT in Men | | |  |
| --- | --- | --- | --- | --- | --- | --- | --- | --- |
|  | **All**  9,841 (100) | **Above median**  4,920 (50) | **Below median**  4,921 (50) | ***P*** | **All**  14,167 (100) | **Above median**  7,083 (50) | **Below median**  7,084 (50) | ***P*** |
| EAT volume |  |  |  |  |  |  |  |  |
| All-cause mortality | 1,532 (15.6) | 877 (17.8) | 655 (13.3) | **<0.001** | 3,136 (22.1) | 1,773 (25.0) | 1,363 (19.2) | **<0.001** |
| Cardiovascular mortality | 323 (3.3) | 187 (3.8) | 136 (2.8) | **0.002** | 760 (5.4) | 456 (6.4) | 304 (4.3) | **<0.001** |
| EAT density |  |  |  |  |  |  |  |  |
| All-cause mortality | 1,532 (15.6) | 707 (14.4) | 825 (16.8) | **0.002** | 3,136 (22.1) | 1,505 (21.2) | 1,631 (23.0) | **0.028** |
| Cardiovascular mortality | 323 (3.3) | 152 (3.1) | 171 (3.5) | 0.286 | 760 (5.4) | 372 (5.3) | 388 (5.5) | 0.573 |

Values are given as the raw number (percentage). All *P*-values are based on unadjusted log-rank tests. Sex-specific EAT volume median was 62.6 cm³/m² in women and 70.8 cm³/m² in men. Sex-specific EAT density median was -77.0 HU in women and -78.0 HU in men. EAT = epicardial adipose tissue.

**TABLE S3. Association of Baseline EAT with Mortality in Women and Men**

|  | Women | | | Men | | | Interaction |
| --- | --- | --- | --- | --- | --- | --- | --- |
|  | **HR** | **95% Cl** | ***P*** | **HR** | **95% Cl** | ***P*** | ***P**** |
| EAT Volume | | | | | | | |
| All-cause Mortality | 1.10 | 1.06–1.15 | **<0.001** | 1.13 | 1.10–1.16 | **<0.001** | 0.945 |
| Cardiovascular Mortality | 1.16 | 1.07–1.26 | **<0.001** | 1.18 | 1.12–1.25 | **<0.001** | 0.713 |
| EAT Density | | | | | | | |
| All-cause Mortality | 1.20 | 0.97–1.47 | 0.090 | 1.41 | 1.22–1.63 | **<0.001** | 0.366 |
| Cardiovascular Mortality | 1.90 | 1.22–2.95 | **0.004** | 1.69 | 1.25–2.27 | **0.001** | 0.996 |

HRs are adjusted for EAT volume and density, CV risk factors (age, sex, race, ethnicity, smoking status [former vs. current], pack-years, history of heart disease, myocardial infarction [MI], or stroke, diabetes, hypertension, education status, body mass index [BMI], coronary artery calcium [CAC] score, as well as technical parameters (slice thickness, tube voltage [kVp], tube current – exposure time product [mAs], signal-to-noise ratio).

Hazard ratios (HR) and 95% confidence intervals (CI) are per 10 cm^3^/m^2^ (EAT volume) or 10 HU (EAT density).

*P** = *P*-value for sex as interaction term.

**TABLE S4. EAT Changes Over 2 Years in Women and Men**

| N=20,661 | Women | | | |  | Men | | | |  |
| --- | --- | --- | --- | --- | --- | --- | --- | --- | --- | --- |
| EAT volume, cm³/m² | **All**  8,424 (100) | **Stable**  4,378 (52) | **Increase**  2,691 (32) | **Decrease**  1,355 (16) | ***P*** | **All**  12,237 (100) | **Stable**  6,504 (53) | **Increase**  3,496 (29) | **Decrease**  2,237 (18) | ***P*** |
| Baseline | 65.4 ± 22.9 | 67.7 ± 22.7 | 59.3 ± 21.1 | 70.5 ± 24.4 | **<0.001** | 73.3 ± 24.7 | 75.5 ± 24.2 | 66.7 ± 23.3 | 77.1 ± 26.2 | **<0.001** |
| Follow-up | 68.0 ± 23.8 | 69.0 ± 23.2 | 71.3 ± 24.8 | 57.9 ± 21.1 | **<0.001** | 75.8 ± 25.9 | 77.1 ± 24.8 | 81.1 ± 27.3 | 63.6 ± 22.6 | **<0.001** |
| EAT change | +2.5 ±10.2 | +1.3 ± 3.5 | +12.0 ± 7.0 | -12.6 ± 9.9 | **<0.001** | +2.5 ± 11.6 | -1.6 ± 4.0 | +14.3 ± 8.4 | -13.5 ± 9.9 | **<0.001** |
| EAT density, HU | **All**  8,424 (100) | **Stable**  4,444 (53) | **Increase**  1,448 (17) | **Decrease**  2,532 (30) | ***P*** | **All**  12,237 (100) | **Stable**  7,122 (58) | **Increase**  2,233 (18) | **Decrease**  2,882 (24) | ***P*** |
| Baseline | -77.1 ± 5.1 | -77.4 ± 4.9 | -78.9 ± 5.2 | -75.7 ± 4.9 | **<0.001** | -77.9 ± 5.2 | -78.2 ± 5.0 | -79.6 ± 5.3 | -75.9 ± 4.8 | **<0.001** |
| Follow-up | -77.7 ± 5.2 | -77.6 ± 4.9 | -75.4 ± 5.1 | -79.4 ± 5.1 | **<0.001** | -78.4 ± 5.3 | -78.5 ± 5.1 | -75.7 ± 5.1 | -80.3 ± 5.0 | **<0.001** |
| EAT change | -0.6 ± 2.8 | -0.2 ± 1.0 | +3.5 ± 2.0 | -3.7 ± 1.6 | **<0.001** | -0.5 ± 3.1 | -0.3 ± 1.2 | +3.9 ± 2.1 | -4.4 ± 1.8 | **<0.001** |

Values are given as mean ± standard deviation. Stable EAT volume ranged from -7.5% to +10.4% in women, and from -7.6% to +11.1% in men. Stable EAT density ranged from -2.5% to +2.3% in women, and from -3.2% to +2.5% in men. EAT = epicardial adipose tissue; HU = Hounsfield Units.

**TABLE S5. Crude Mortality Rates Across Categories of EAT Change in Women and Men**

|  | EAT changes in Women | | | |  | EAT changes in Men | | | |  |
| --- | --- | --- | --- | --- | --- | --- | --- | --- | --- | --- |
| EAT volume | **All**  8,424 (100) | **Stable**  4,378 (52) | **Increase**  2,691 (32) | **Decrease**  1,355 (16) | ***P*** | **All**  12,237 (100) | **Stable**  6,504 (53) | **Increase**  3,496 (29) | **Decrease**  2,237 (18) | ***P*** |
| All-cause mortality | 1,132 (13.4) | 544 (12.4) | 341 (12.7) | 247 (18.2) | **<0.001** | 2,351 (19.2) | 1,158 (17.8) | 692 (19.8) | 501 (22.4) | **<0.001** |
| Cardiovascular mortality | 245 (2.9) | 132 (3.0) | 58 (2.2) | 55 (4.1) | **0.002** | 571 (4.7) | 291 (4.5) | 162 (4.6) | 118 (5.3) | 0.258 |
| EAT density | **All**  8,424 (100) | **Stable**  4,444 (53) | **Increase**  1,448 (17) | **Decrease**  2,532 (30) | ***P*** | **All**  12,237 (100) | **Stable**  7,122 (58) | **Increase**  2,233 (18) | **Decrease**  2,882 (24) | ***P*** |
| All-cause mortality | 1,132 (13.4) | 519 (11.7) | 259 (17.9) | 354 (14.0) | **<0.001** | 2,351 (19.2) | 1,267 (17.8) | 482 (21.6) | 602 (20.9) | **<0.001** |
| Cardiovascular mortality | 245 (2.9) | 109 (2.5) | 67 (4.6) | 69 (2.7) | **<0.001** | 571 (4.7) | 321 (4.5) | 110 (4.9) | 140 (4.9) | 0.530 |

Values are given as the raw number (percentage). All *P*-values are based on unadjusted log-rank tests, including all three categories of EAT changes (stable, increase, decrease). Stable EAT volume ranged from -7.5% to +10.4% in women, and from -7.6% to +11.1% in men. Stable EAT density ranged from -2.5% to +2.3%, and from -3.2% to +2.5% in men. EAT = epicardial adipose tissue.
